# Mental health in myasthenia gravis patients and its impact on caregiver burden

**DOI:** 10.1038/s41598-022-22078-3

**Published:** 2022-11-11

**Authors:** Derin Marbin, Sophie K. Piper, Sophie Lehnerer, Ulrike Harms, Andreas Meisel

**Affiliations:** 1 Present Address: Department of Neurology with Experimental Neurology, Charité – Universitätsmedizin Berlin, Berlin, Germany; 2 Psychiatrische Universitätsklinik der Charité Im St. Hedwig-Krankenhaus, Berlin, Germany; 3 Institute of Biometry and Clinical Epidemiology, Charité – Universitätsmedizin Berlin, Corporate member of Freie Universität Berlin and Humboldt - Universität Zu Berlin, Charitéplatz 1, 10117 Berlin, Germany; 4 NeuroCure Clinical Research Center, Charité – Universitätsmedizin Berlin, Berlin, Germany; 5 Center for Stroke Research Berlin, Charité – Universitätsmedizin Berlin, Berlin, Germany

**Keywords:** Neurology, Psychology

## Abstract

Psychiatric comorbidities are relevant in patients with Myasthenia gravis (MG). Also, MG patients experience a reduced health-related quality of life (HRQoL). We aimed to quantify the impact of depression as well as self-perceived MG severity on HRQoL and caregivers' burden. In this cross-sectional study, we used a survey encompassing demographic, disease-related information, and standardized questionnaires to assess symptoms of depression, anxiety, HRQoL (MG Quality of Life scale; MG-QoL15), and caregiver burden (Burden Scale for Family Caregivers; BSFC). Data from 1399 participating patients (96%) and 1042 caregivers (65%) were eligible for further analysis. Symptoms of depression and anxiety disorder were indicated by 31% and 36% of patients. Self-reported MG severity (MG severity) and MG-QoL15 scores were strongly associated (estimated marginal means for severe versus mild MG severity = 18 95% CI [16; 21]; *p* ≤ 0.001). Adjusting for symptoms of depression decreased the estimated strength of this association (estimated marginal means for severe versus mild MG severity = 15 [13; 17]; *p* ≤ 0.001). Caregiver burden was associated to MG disease severity (estimated marginal means for severe vs. mild MG severity = 0.16 [0.13; 0,19); *p* ≤ 0.001) and also negatively influenced by symptoms of depression (estimated marginal means = 0.12 [0.09; 0.15]). Symptoms of depression and anxiety disorder in MG are frequent. Beyond MG severity, symptoms of depression have negative effects on HRQoL as well as on caregivers’ burden. Diagnosis and treatment of psychiatric comorbidities should be considered an important element in MG care. Screening tools for mental health conditions should be implemented at least in specialized MG centers.

## Introduction

Myasthenia gravis (MG) is a chronic, autoimmune-mediated disease of the neuromuscular junction with fluctuating weakness in skeletal muscles^1^. The median prevalence is 15 per 100,000 persons^2^. Symptoms can encompass ocular such as diplopia and ptosis, faciopharyngeal weakness like dysphagia and dysarthrophonia as well as generalized weakness affecting limb, neck, and torso musculature. Almost 20% of patients suffer from a life-threatening myasthenic crisis at least once during the disease. It is characterized by respiratory insufficiency, requiring intensive care treatment with mechanical ventilation^3,4^. Although significant advances in therapy prolonged overall life expectancy, a reduced Health-Related Quality of Life (HRQoL) is reported, especially among MG patients with bulbar and generalized symptoms^5^. There is inconsistent data about the reduction of psychological domains in the HRQoL^5,6^. Activities of daily life (AdL) are also reduced in MG patients^7^. The impact of MG itself as well as its comorbidities including those affecting mental health have become an important focus in research.

Psychiatric comorbidities like depression, anxiety disorder, or posttraumatic stress disorder (PTSD) are highly prevalent in smaller cohorts of MG patients, with prevalences of depression ranging from 14 to 58%^8,9^. Depression and other factors like illness stability and impairments have been identified as important factors affecting the HRQoL^10^, which needs to be considered in the MG patients’ treatment^11^. So far, the detection and adequate treatment of mood disorders in MG patients seems insufficient^12,13^. However, the impact of MG severity and related mental disorders on the burden of disease is limited. Importantly, their effects on caregivers' burden are unknown. We hypothesize that the prevalence of depression, anxiety disorder, and PTSD is not only higher amongst MG patients, but also underdiagnosed. We further hypothesize that the self-perceived MG severity, as well as comorbid depression, have a significant impact on patients’ HRQoL and caregivers’ burden.

Thus, we investigate the prevalence and possible underdiagnosis of psychiatric symptoms (depression, anxiety, and PTSD) in MG patients. We aim to quantify the effect of MG severity and symptoms of depression on patients' HRQoL and caregivers' burden.

## Methods

### Patients

In cooperation with the German Myasthenia Society (self-help group for MG in Germany; DMG), we reached out to all 3280 members via postal mail between April and October 2017 asking them to fill out a questionnaire.

The study participants were instructed to return their completed questionnaire in a prepaid envelope with no identifying information to ensure the anonymity of the survey and its participants. Further patients were recruited by our physicians in our MG outpatient clinic (“Integrated MG Center”) at Charité University Hospital, Berlin. Prior to enrollment, all patients had to confirm that they did not already participate in this study via the DMG and gave written informed consent. No refund was given.

All participants were asked to encourage their caregivers to provide information via an additional questionnaire assessing the caregiver burden. Exclusion criteria were participants’ age under 18, missing diagnosis of MG, and insufficiently completed questionnaires, defined a priori in case less than fifty percent of all items answered. All data was de-identified for analysis. The study has been approved by the Charité University Medicine Berlin’s ethics committee (reference number EA1/028/17) and registered at www.clinicaltrials.gov (NCT03205306). All research was performed following the Helsinki Declaration.

### Parameters and scores

The questionnaires contained demographic data such as gender, age, marital status, and educational status. Relevant disease-related information such as current disease severity (low, medium, high), antibody status, age at onset of myasthenia-related symptoms and time of diagnosis, comorbidities (autoimmune, cardiac or psychiatric (depression, anxiety disorder, PTSD, other)), medication, history of myasthenic crisis and exacerbation.

Psychiatric symptoms and burden of disease were assessed by using the following validated German versions of standardized and self-administrated questionnaires: Hospital Anxiety and Depression Scale (HADS), MG Quality of Life Scale (MG-QoL15), short DSM-IV screening scale for PTSD (PTSD-7), Freiburger Fragebogen zur Krankheitsverarbeitung (FKV; not available in English) and Burden Scale for Family Caregivers (BSFC).

The HADS is a screening instrument with high validity and reliability (sensitivity and specificity 0.8; Cronbach’s alpha for Anxiety: 0.83 and for Depression: 0.82) to detect depression and anxiety amongst patients with somatic diseases^14^. It contains seven items for each anxiety and depression. The cut-off score is seven, indicating cases above cut-off in gradation mild (8–10), moderate (11–14), and severe (> 14). The MG-QOL15 is a short version of the sixty items MGQOL^15^. It is a disease-specific health-related QoL scale, with lower scores indicating a higher HRQoL.

We asked each patient with a myasthenic crisis (with ICU submission) or exacerbation throughout the disease to fill out the PTSD-7^16,17^. A sum score of ≥ 4 implies the possibility of PTSD with a sensitivity of 80% and specificity of 97%, and reliability is also high (Cronbach’s alpha 0.90)^16^. To investigate coping strategies, we used the FKV questionnaire, assessing different dimensions of coping mechanisms, e.g. cognition, emotion, and behavior^18^.

To examine perceived caregivers' burden, we used the short version of the BSFC including 10 domains such as reduced life satisfaction, physical exhaustion, conflicting demands, or affection for their health by caregiving. Cronbach's alpha for the complete scale was 0.92^19^.

### Statistical analysis

For data transfer and analysis, we used *IBM SPSS Statistics 25* and *STATA IC 14*. We compared sociodemographic characteristics, questionnaire scores, and clinical characteristics between the myasthenia subgroups (disease severity) using the chi‐square test, or Kruskal–Wallis-test, where indicated.

General linear models were used to estimate the association between self-reported disease severity as the independent variable and MG-QoL15 and caregiver burden as dependent variables, respectively. Models were stepwise adjusted for potential effect mediation by depression according to HADS scores (1: < 8; 2: 8–10; 3: 11–14; 4: > 14), as well as the potential confounding factors gender, age, education, marital status, myasthenic crisis/exacerbation, duration of disease, disease latency and treatment escalation (IVIG/Rituximab) as well as a history of other autoimmune disease or cardiovascular disease as binary variables.

Continuous variables with a right-skewed distribution, namely the duration of disease and the disease latency were log-transformed before being entered into the model. Adjusted R^2^ values are given for goodness of fit. A two-sided significance level of α = 0.05 was considered. No adjustment for multiple testing was applied and all p-values constitute exploratory data analysis.

### Ethics approval

The study has been approved by the Charité University Medicine Berlin’s ethics committee (reference number EA1/028/17), and registered at www.clinicaltrials.gov (NCT03205306).

### Consent to participate

Informed consent was obtained from all individual participants included in this study.

## Results

The questionnaires were filled out by 1451 patients yielding an overall response rate of 44.2% (Table 1). Considering to the exclusion criteria, 52 questionnaires had to be excluded. Caregivers’ response rate was 47% (1596). Incompletely filled-out questionnaires had to be excluded, 65% of the questionnaires were eligible for further analyses (1042). The median duration of MG was 9 years (IQR 4–18), the median age of MG onset was 39 years (IQR 24–57) in women and 62 years (IQR 51–70) in men. Correspondingly, the median age of diagnosis was 44 years (IQR 28–60) in women and 62 years (IQR 51–70) in men. Less than half of the patients had undergone thymectomy (42.9%), with 67.7% of them being female patients. Intake of regular and delayed-release pyridostigmine was reported by 63.5% and 39.0%. The most commonly used immunosuppressant was azathioprine (43.8%), and steroids were taken by 23.4% of the participants. Escalation therapy with Rituximab and IVIG was reported by 3.4% and 8.2%, respectively. Experience of at least one myasthenic crisis was reported by 17.2% of patients, whereas 22.8% reported exacerbations of MG. The lead institution in MG care was a certified center for MG care ("integrated myasthenia center") for 20.7% of patients and a neurological outpatient clinic of a university hospital for 30.9%. The majority of patients (60.8%) also visited a registered neurologist or general practitioner regularly. The most abundant somatic comorbidities, cardiovascular and autoimmune disorders were reported by 18.1% and 18.9%, respectively (Table 1), with rheumatoid arthritis, hypothyroidism, Hashimoto’s thyroiditis, psoriasis, and systemic lupus erythematosus being the most often reported autoimmune disorders (data not shown).

**Table 1:** Characteristics of MG patients.

	Total	Low	Moderate	High	*p*-value
Self-reported MG severity, n (%) n = 1329	1399	469 (35.3)	690 (51.9)	170 (12.8)	
Age, median (IQR). n = 1329	67 (54–76)	66 (54–75)	66 (54–76)	68 (55–77)	0.376^a^
**Age at onset of myasthenic symptoms, median (IQR). n = 1257**					
Male	62 (51–70)	61 (51–69)	63 (52–70)	60,5 (49–70)	0.498 ^a^
Female	39 (24–57)	37 (24–55)	41 (25–58)	34 (20–56)	0.148 ^a^
**Gender n = 1327**					
Male	605 (45.6)	248 (53.1)	280 (40.6)	77 (45.3)	**< 0.001**^**b**^
Female	722 (54.4)	219 (46.9)	410 (59.4)	93 (54.7)	
**Education degree n = 1245**					
Basic	62 (5.0)	19 (4.3)	33 (5.2)	10 (6.2)	**0.014**^**a**^
Secondary	778 (62.5)	260 (58.4)	422 (66.1)	96 (59.3)	
Higher	405 (32.5)	166 (37.3)	183 (28.7)	56 (34.6)	
**Marital status n = 1325**					
Married/partnership	986 (74.4)	358 (76.3)	497 (72.3)	131(77.5)	0.225^a^
Single	106 (8.0)	38 (8.1)	61 (8.9)	7 (4.1)	
Divorced/widowed	233 (17.6)	73 (15.6)	129 (18.8)	31 (18.3)	
Duration of MG disease in years, median (IQR). n = 1329	9 (4–18)	10 (4–18)	9 (4–18)	11 (5–22)	**0.041**^**a**^
Latency of diagnosis in years, median (IQR). n = 1250	0 (0–1)	0 (0–1)	0 (0–2)	0 (0–2)	**< 0.001**^**a**^
Myasthenic crisis. n = 1384	240 (17.2)	44 (9.4)	116 (16.8)	67 (39.4)	**< 0.001**^**b**^
Myasthenic exacerbation. n = 1384	319 (22.8)	79 (16.8)	194 (28.1)	46 (27.1)	**< 0.001**^**b**^
Other autoimmune diseases n = 1280	264 (18.9)	81 (17.3)	155 (22.5)	28 (16.5)	**0.047**^**b**^
Cardiovascular diseases. n = 1289	253 (18.1)	72 (15.4)	139 (20.1)	42 (24.7)	**0.010**^**b**^
Thymectomy. n = 1302	600 (42.9)	212 (45.2)	303 (44.0)	85 (50.0)	0.295^b^
Pyridostigmine. n = 1328	888 (63.5)	253 (54.0)	505 (73.2)	130 (76.5)	**< 0.001**^**b**^
Delayed-release Pyridostigmine retard. n = 1329	545 (39.0)	125 (26.7)	341 (49.4)	79 (46.5)	**< 0.001**^**b**^
Azathioprine. n = 1329	613 (43.8)	194 (41.4)	360 (52.2)	59 (34.7)	**< 0.001**^**b**^
Steroids. n = 1251	328 (23.4)	71 (15.2)	193 (28.0)	64 (37.6)	**< 0.001**^**b**^
Mycophenolate Mofetil. n = 1329	126 (9.0)	27 (5.8)	68 (9.9)	31 (18.2)	**< 0.001**^**b**^
Methotrexate. n = 1329	53 (3.8)	21 (4.5)	20 (2.9)	12 (7.1)	0.037^b^
Ciclosporine A. n = 1329	15 (1.1)	0 (0.0)	12 (1.7)	3 (1.8)	**0.016**^**b**^
Rituximab. n = 1329	47 (3.4)	5 (1.1)	21 (3.0)	21 (12.4)	**< 0.001**^**b**^
Intravenous immunoglobulin. n = 1329	115 (8.2)	11 (2.3)	57 (8.3)	47 (27.6)	**< 0.001**^**b**^
MGQOL15 score, median (IQR). n = 1037	12 (4–26)	4 (1–10)	18 (9–29)	25 (9–385)	**< 0.001**^**a**^
Sleep difficulties. n = 1183	881 (63.0)	247 (52.7)	512 (75.2)	122 (71.8)	**< 0.001**^**b**^
Sexual dysfunction. n = 1209	755 (54.0)	201 (42.9)	442 (64.1)	112 (65.9)	**< 0.001**^**b**^
BSFC score, median (IQR). n = 977	3 (0–10)	1 (0–4)	4 (0–12)	9 (2–15)	**< 0.001**^**a**^

N = 1399.

*ICU *Intensive care unit

P-values refer to overall group comparisons of patients with low, moderate and high MG severity. *P*-values < 0.05 are marked in bold numbers.

^a^Kruskal-Wallis-Test, ^b^Chi-square test.

Of all, 81.4% negated a diagnosed mental health condition, a prediagnosed depression has been reported by 11.6%, and 5.7% reported an anxiety disorder (Table 2). In contrast, when assessing the prevalences of psychiatric symptoms by using standardized questionnaires (HADS), we observed significantly higher rates of symptoms of depression (30.8%) and anxiety disorder (35.5%). Signs of possible PTSD could be identified in 10.7% of patients with a self-reported myasthenic crisis (with ICU submission) or exacerbation, which equals 4.4% of the total study population. There were no differences in the frequencies of possible PTSD between patient groups reporting myasthenic crisis and exacerbation. The severity of symptoms of depression as well as anxiety disorder was associated significantly with higher MG severity. The numbers of symptoms of depression and possible PTSD was almost three to eight times higher in patients with high MG severity compared to low (39.0% vs. 14.4% in symptoms of depression; 8.2% vs. 1.1% in PTSD). Amongst MG patients with high MG severity, anxiety was twice as common as in patients with low disease severity (38.4% vs. 19.4%).

**Table 2:** Psychiatric comorbidities in MG patients.

	missing	Total	Low	Moderate	High	*p*-value
Self-reported MG severity, n (%)		1399	469 (33.5)	690 (51.9)	170 (12.8)	
**Depression (HADS)**						
No depression	21	957 (69.2)	392 (85.6)	407 (60.1)	100 (61.0)	**< 0.001**^a^
Depression		421 (30.8)	66 (14.4)	270 (39.9)	64 (39.0)	
Mild		217 (15.7)	38 (8.3)	142 (21.0)	27 (16.5)	
Moderate		160 (11.6)	23 (5.0)	113 (16.7)	34 (20.7)	
Severe		49 (3.5)	5 (1.1)	15 (2.2)	3 (1.8)	
Prediagnosed depression	21	162 (11.6)	35 (7.5)	101 (14.6)	21 (12.4)	**0.001**^**b**^
Pharmacotherapy^e^	8	92 (56.0)	20 (57.0)	58 (57.0)	14 (67.0)	
Psychotherapy^e^	28	47 (29.0)	13 (37.0)	31 (31.0)	3 (14.0)	
**Anxiety (HADS)**						
No anxiety	22	903 (65.6)	369 (80.6)	379 (56.0)	101 (61.6)	**< 0.001**^a^
Anxiety		474 (35.5)	89 (19.4)	299 (44.0)	63 (38.4)	
Mild		259 (18.8)	58 (12.7)	158 (23.3)	28 (17.1)	
Moderate		162 (11.8)	29 (6.3)	118 (17.4)	27 (16.5)	
Severe		53 (3.8)	2 (0.4)	23 (3.2)	8 (4.9)	
Prediagnosed anxiety	24	80 (5.7)	14 (3.0)	52 (7.5)	12 (7.1)	**0.004**^b^
Pharmacotherapy^e^	397	45 (56.0)	9 (64.0)	29 (56.0)	17 (24.0)	
Psychotherapy^e^	415	21 (26.0)	3 (21.0)	17 (33.0)	1 (1.0)	
PTSD-7 after myasthenic crisis	62	61 (10.7) (4.4% of the total)	5 (1.1)	42 (6.1)	14 (8.2)	**0.020**^a^
Prediagnosed PTSD	41	62 (10.8)	11 (2.4)	39 (5.7)	12 (7.0)	**0.001**^b^

*PTSD *Posttraumatic stress disorder.

*P*-values refer to overall group comparisons of patients with low, moderate and high MG severity. *P*-values < 0.05 are marked in bold numbers

^a^Kruskal–Wallis-Test, ^b^Chi-square test.^*e*^ % of prediagnosed cases.

A third of patients with a psychiatric diagnosis were undergoing psychotherapeutic treatment (35.5%), and more than half were undergoing pharmacotherapy (57.8%). Antidepressants were taken by 69.2% of patients with severe and 64.8% of patients with moderate depression according to HADS. In contrast, only 50.0% with severe and 36.2% with moderate symptoms of depression were in psychotherapy. Importantly, the majority of patients who underwent psychotherapy (93.9%) reported benefiting therefrom. Sleep difficulties were reported by 63.0%. A restricted sexual life due to their MG was reported by 54.0% of patients.

The median MG-QoL15 score was 12 (IQR 4–26; Table 1). Linear regression analysis revealed that the self-reported MG severity was strongly associated with higher MG-QoL15 scores. The estimated marginal means for MG-QoL15 score for severe vs. mild self-reported MG severity was 18.26 95%CI [15.92; 20.60]; Table 3), suggesting a lower HRQoL in patients with a high MG severity as compared to those with mild MG severity (adj R^2^ = 0.264).

**Table 3:** Associations between self-reported MG severity and MGQOL15 *scale scores* (z-transformed continuous outcome).

	M1: self-reported severity N=1053	M2: additionally adjusted for depression N=1049	M3: additionally adjusted for gender N= 1049	M4: additionally adjusted for other variables^a^ N= 975
**Self-reported MG severity**				
Moderate vs. mild	13.16 (11.63–14.69)	9.40 (8.07–10.72)	8.99 (7.68–10.30)	8.09 (6.75–9.44)
Severe vs. mild	18.26 (15.92–20.60)	15.37 (13.39–17.35)	15.09 (13.15–17.04)	14.36 (12.23–16.48)
**Depression (HADS)**				
mild vs. healthy*	–	11.56 (9.85–13.27)	11.67 (9.98–13.35)	11.51 (9.78–13.23)
moderate vs. healthy	–	16.81 (14.79–18.83)	16.82 (14.83–18.81)	17.00 (15.01–19.00)
severe vs. healthy	–	21.37 (17.71–25.02)	21.69 (18.10–25.29)	21.05 (17.33–24.77)
**gender**				
Female vs. male	–	–	3.54 (2.36–4.72)	2.86 (1.48–4.24)
Other autoimmune disorder				1.58 (0.11–3.05)
Rituximab or IVIG intake				3.83 (1.83–5.84)
thymectomy				− 0.85 (− 2.23–0.51)
Adj R^2^	0,264	0,490	0,506	0,560

Stepwise Linear regression analysis. Estimated marginal means with 95%CI for MGQOL15 scale scores. *healthy = no depression according to HADS.

^a^M4: Additionally adjusted for thymectomy, other autoimmune disease, cardiovascular disease, IVIG/Rituximab, myasthenic crisis/exacerbation, duration, disease latency age, education, marital status.

**Figure 1:**
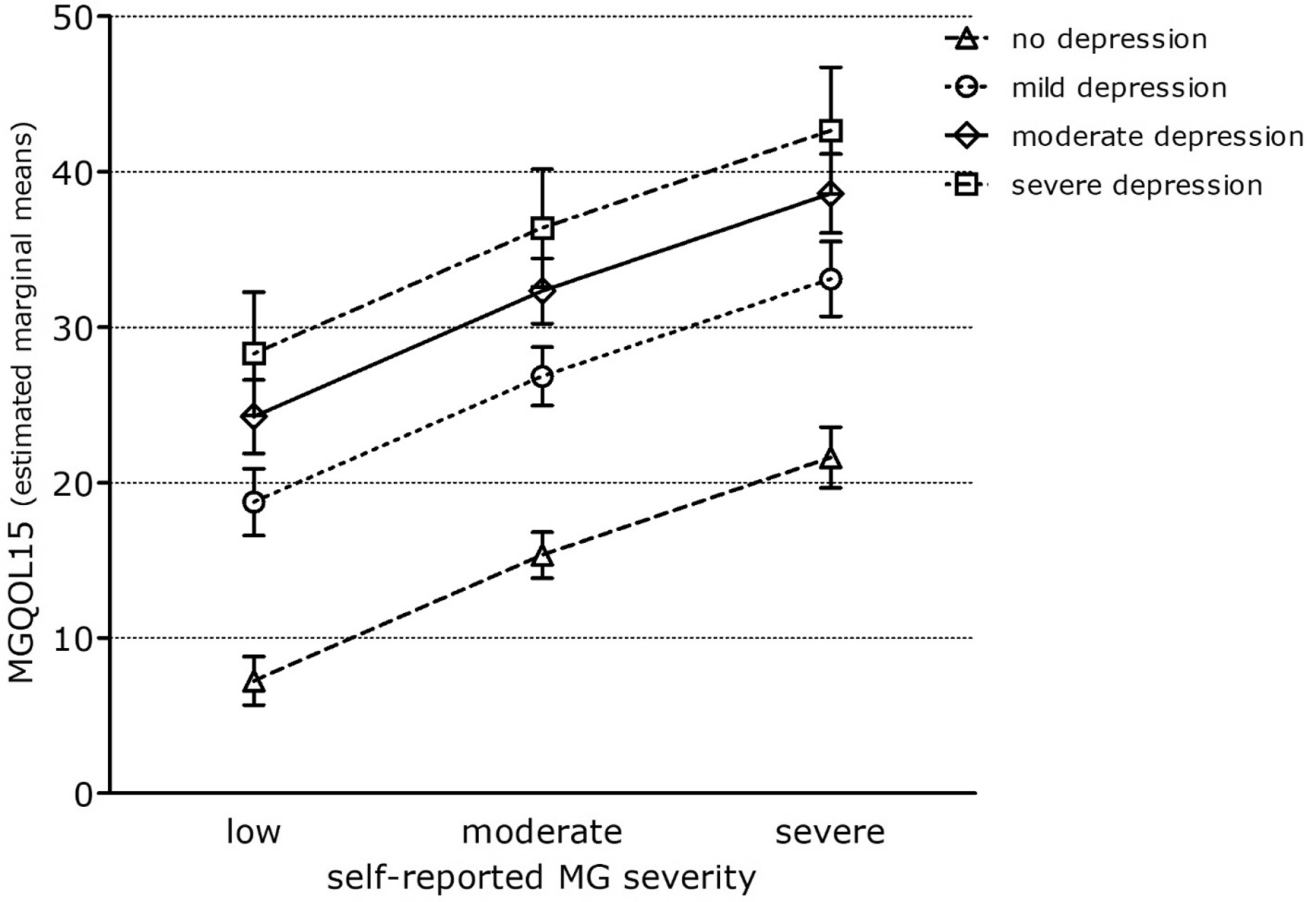
Adjusted MGQOL15 scale scores for different severities of depression according to HADS. Estimated marginal means with 95%CI for MGQOL15 scale scores after full adjustment for depression, gender, thymectomy, other autoimmune disease, cardiovascular disease, IVIG/Rituximab, myasthenic crisis/exacerbation, duration, disease latency age, education, marital status. MGQOL15 = Myasthenia gravis Quality of Life Score, short version; HADS = Hospital Anxiety and Depression Scale.

The estimated strength of association for MG severity on MG-QoL15 decreased after adjusting for symptoms of depression, whereas model fit increased. The estimated marginal means of MG-QoL15 score for severe vs. mild MG severity = 15.37 95%CI [13.39; 17.35]; Adj R^2^ = 0.490; Table 3). Depression as a possible comorbidity might partially explain the association between MG disease severity and HRQoL. Accordingly, in model 2, higher depression categories were significantly associated with higher MG-QoL15 scores, therefore with a decreased HRQoL (Fig. 1).

In contrast, adjustment for gender in model 3 as well as for age, education, marital status, myasthenic crisis, duration of disease, disease latency, and treatment escalation (IVIG/rituximab) in model 4 only slightly affected the effect estimates of self-reported severity (estimated marginal means of MG-QoL15 score for severe vs. mild MG severity = 14.36 95%CI [12.23 16.48]; Adj R^2^ = 0.560; Table 3). In model 4, the female gender was significantly associated with a lower HRQoL (higher MG-QoL15 score values; estimated marginal means of MG-QoL15 scale score for female vs. male patients = 2.86 95%CI [1.48; 4.24]). The same was observed for escalation therapy with IVIG or Rituximab (estimated marginal means of MG-QoL15 scale score for escalation therapy vs. no escalation therapy = 3.83 95%CI [1.83; 5.84]). In contrast, thymectomy was associated with a higher HRQoL (lower MG-QoL15 score values; estimated marginal means for MG-QoL15 scale score for thymectomy vs. no thymectomy = − 0.85 95%CI [− 2.23; − 0.51]).

**Figure 2:**
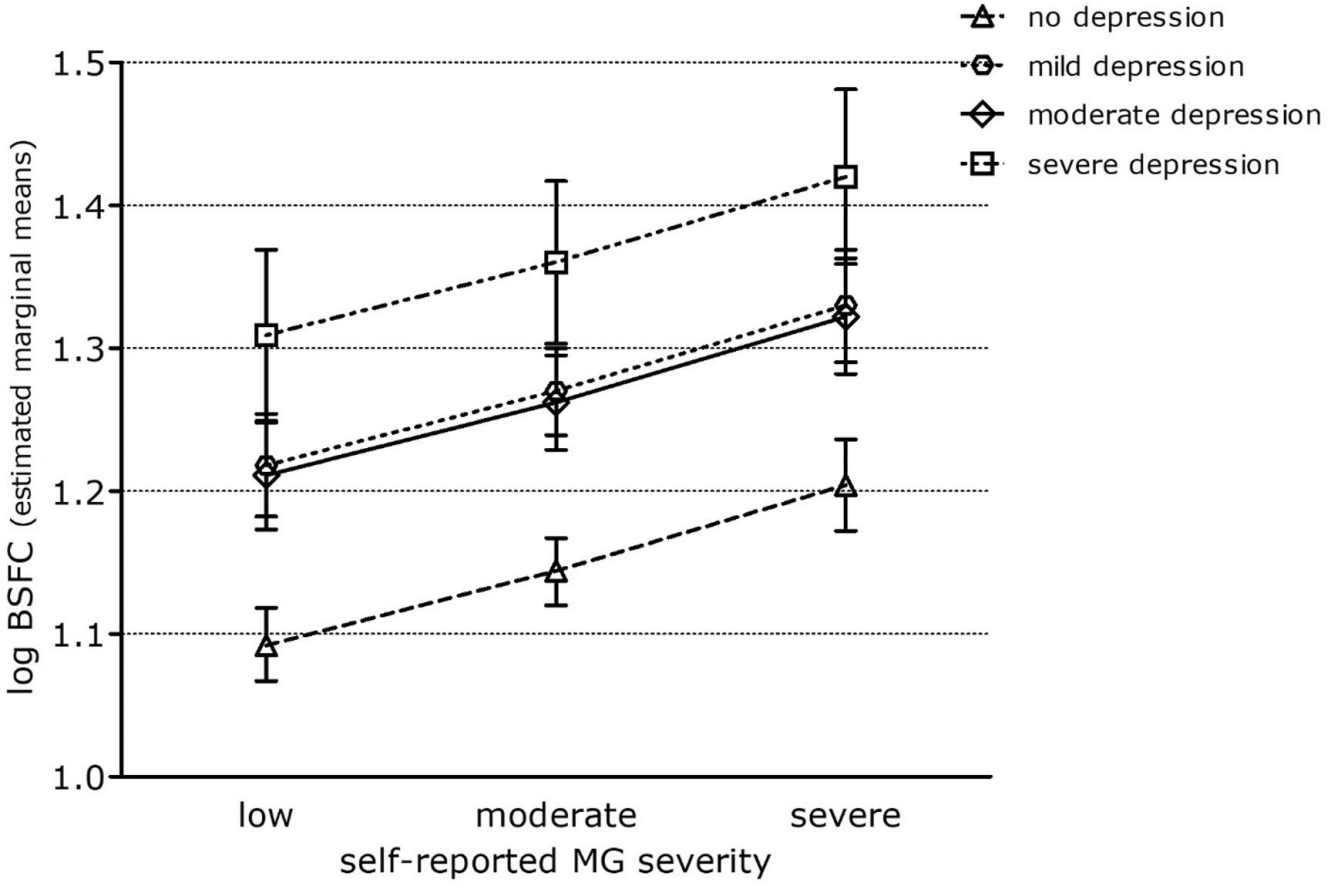
Adjusted logarithmized BSFC scale scores for different categories of depression according to HADS. Estimated marginal means with 95%CI for logarithmized BSFC scale scores after full adjustment for depression, gender, thymectomy, other autoimmune disease, cardiovascular disease, IVIG/Rituximab, myasthenic crisis/exacerbation, duration, disease latency age, education, marital status. BSFC = burden scale for family caregivers; HADS = Hospital Anxiety and Depression Scale.

The MG severity was associated with the caregiver burden as measured by the BSFC (estimated marginal means of logarithmized BSFC scale score for severe vs. mild MG severity = 0.16 95%CI [0.13; − 0.19)]; Adj R^2^ = 0.10; Table 4). Again, the effect estimates for MG severity decreased after adjustment for depression according to HADS (estimated marginal means of logarithmized BSFC scale score for severe vs. mild symptoms of depression = 0.12 95%CI [0.09; 0.15]; Adj R^2^ = 0.22), suggesting that depression, as possible comorbidity of the patient, explained in part the association between MG severity and caregiver burden (adj R^2^ = 0.223). In model 2, higher depression categories were significantly associated with higher BSFC scale scores (Fig. 2).

**Table 4:** Associations between self-reported MG severity and BSFC scale scores (log-transformed continuous outcome).

	M1: self-reported severity N= 997	M2: additionally adjusted for depression N= 991	M3: additionally adjusted for gender N= 989	M4: additionally adjusted for other variables^a^ N= 907
**Self-reported MG severity**				
Moderate vs. mild	0.09 (0.07–0.11)	0.06 (0.04–0.08)	0.06 (0.04–0.08)	0.05 (0.03–0.07)
Severe vs. mild	0.16 (0.13–0.19)	0.12 (0.09–0.15)	0.12 (0.09–0.15)	0.11 (0.08–0.15)
**Depression (HADS)**				
Mild vs. healthy*	–	0.12 (0.09–0.14)	0.12 (0.09–0.14)	0.13 (0.10–0.15)
Moderate vs. healthy	–	0.12 (0.10–0.15)	0.13 (0.10–0.16)	0.12 (0.09–0.15)
Severe vs. healthy	–	0.22 (0.17–0.28)	0.22 (0.17–0.27)	0.22 (0.16–0.27)
**Gender**				
Female vs. male	–	–	− 0.03 (− 0.50 to − 0.01)	− 0.03 (− 0.05 to − 0.01)
Thymectomy				− 0,02 (− 0,04–0.00))
Age				− 0,00 (− 0,01–0,00)
Adj R^2^	0,098	0,224	0,232	0,265

Stepwise linear regression analysis. Estimated marginal means with 95%CI for log-transformed BSFC scale scores. *healthy = no depression according to HADS. ^a^M4: Additionally adjusted for thymectomy, autoimmune disease, cardiovascular disease, IVIG/Rituximab, myasthenic crisis/exacerbation, duration, disease latency, age, education, marital status.

Adjusting for gender in model 3 and for the other potentially confounding variables in model 4 showed no substantial change in effect estimates, suggesting that these factors did not have an impact on the association between MG severity and caregiver burden (Table 4), except thymectomy having a minor impact.

## Discussion

This study demonstrates that symptoms of depression, anxiety disorder, and PTSD in MG patients were not only high among MG patients but that, with implications of higher prevalences of depression and anxiety disorder, these conditions might also be underdiagnosed. Out of all MG patients, 81.4% reported no diagnosed mental health condition. Patients with higher MG severity reported depression and anxiety disorder more frequently than patients with lower MG severity. MG disease severity was strongly associated with patients' HRQoL as well as caregiver burden. Beyond MG severity, symptoms of depression had negative effects on HRQoL in MG patients as well as on caregiver burden. In addition to the effect of depression, patients' HRQoL as well as caregiver burden were negatively associated with the patient’s female gender and positively associated with thymectomy.

In the literature so far, the prevalence of depression in MG patients differed between 14 and 58%^9,20^, whereas the prevalence of anxiety disorders ranged between 20 and 55%^21–23^,. This variability can be explained by the considerable heterogeneity of the cohorts, sample sizes, clinical measures, and assessment methods, including the diagnostic criteria used. PTSD has been reported in 51% of MG patients after an episode of respiratory insufficiency. A positive finding in PTSD screening was more likely in patients with higher HADS score values^24^. The prevalence of psychiatric comorbidities in the general population in Germany^25^ is much lower when compared to this study’s findings of related symptoms in the MG cohort data (15.4% vs. 35.5% for anxiety; 8.2% vs. 30.8% for symptoms of depression, and 2.3% vs. 4.9% for PTSD). Interestingly, the PTSD-7 results revealed similar rates of PTSD in MG patients with a history of an exacerbation and a myasthenic crisis. Compared to the prevalence of anxiety disorder of 24% to 29% in post-stroke and 22% in multiple sclerosis patients, MG patients were substantially affected more often^26–28^.

Our data based on the HADS questionnaire suggests an underdiagnosis of depression and anxiety disorder MG patients. MG patients with symptoms of depression and anxiety disorder had a corresponding diagnosis in only 23.3% and 12.7%, respectively.

In the largest study to date which has been conducted prior to 2010, 38.6% of 1518 patients had a diagnosed depression^10^, the rates were three times higher than in our study but close to the results of HADS. While the other characteristics of the MG patients were very similar to our study with both being conducted in Germany, the difference is likely due to a changed situation in MG patients’ care (previously combined specialty for neurology and psychiatry with now often separated residency tracks). Our study highlights the importance of increasing the awareness of mental health conditions and the necessity for implementing diagnostic measures, such as easy-to-use screening tools. These tools might help to diagnose and treat comorbid mental health conditions in MG patients more effectively. For example, studies have shown that stroke patients benefit from screening for depression^29^.

The simultaneous presence of MG and symptoms of depression, for instance, can be challenging for diagnosing depression in MG patients and vice versa. Thus, depressive symptoms can be misinterpreted as myasthenic symptoms such as fatigue and the other way around. In contrast, the difficulty in clearly distinguishing symptoms of depression from fatigability and fatigue symptoms of MG might lead to delayed or misdiagnosis in MG patients^30^. It has been reported that only one patient out of ten with myasthenic symptoms gets an adequate MG diagnosis^31^. Young women are more likely to get a psychiatric diagnosis, whereas men are more often misdiagnosed with other somatic diseases^32^. This is consistent with our study’s findings that women have significantly higher latencies in diagnosis than men (3.0 vs. 1.3 years). This not only raises the question of why women are diagnosed later but also whether a higher latency of diagnosis increases the risk for depression in women. The overlap of myasthenic and depressive symptoms in MG patients (with or without comorbid depression) leads to difficulties for physicians in making an adequate diagnosis. We demonstrated a strong association between self-reported disease severity and HRQoL, which is largely affected by comorbid depression. This is consistent with the results of a previous study of eighty patients^33^. Our results suggest that depressive symptoms are—amongst the variables we investigated—the most influential factor affecting the perceived MG severity and HRQoL. We cannot rule out that the effect is at least partially inverse, with higher MG severity and lower HRQoL leading to a higher likelihood to develop depressive symptoms. The female gender was strongly associated with lower HRQoL corroborating findings of previous studies^5,34^. It has also been reported that the female gender as well as depression are directly associated with worse disease severity^35,36^. Several factors might explain the gender differences in HRQoL, including differences in experiencing and reporting the severity of symptoms in general. Female patients tend to report significantly more physical symptoms and higher symptom severity levels than men^37^, which might reduce the HRQoL as shown by our results. Secondly, according to our data female patients experienced a long latency between the onset of symptoms and diagnosis. During this period, circumstances like physician hopping, inadequate therapies, the experience of discrimination and humiliation, and misdiagnosis due to broad differential diagnoses might be stressful and therefore affect the HRQoL. The understanding of the interaction of depression, gender, perceived disease severity, and HRQoL in MG patients is scarce. A recent study with 179 patients showed a moderate correlation between disease severity and depression as well as the female gender^35^. For other chronic diseases like chronic renal disease, rheumatoid arthritis, and cardiovascular disease depression is a known factor affecting perceived disease severity^38–40^.

We observed an improved HRQoL after thymectomy. Consistent with our results, gender differences in HRQoL were abolished in MG patients after thymectomy^34^. The burden of disease was lower after thymectomy, and patients had fewer exacerbations and also required less immunosuppressive medication^41,42^.

MG disease severity and the caregiver burden were strongly associated. Symptoms of depression increased the effect of this association, but it also had a direct and negative effect on the caregiver burden. In the general population, the mental status of a care recipient was associated with the caregiver burden and, more importantly, the caregiver burden was nearly significant in predicting depression in caregivers^43^. Thus, another important aspect of MG care should be the provision of supportive measures not only for patients but also for their caregivers. For example, when caregivers are under greater strain, outpatient care services, and psychological assistance, especially with systemic therapy, psycho-education, and self-help groups can provide relief to the social tension field of families. Female patients’ caregivers showed a lower caregiver burden, whereas female caregivers of male MG patients experienced a higher burden. In general, female caregivers experience a more significant caregiver burden than men^44^. The higher strain has been explained by women experiencing more secondary stressors, such as financial and relational problems. Depending on the cultural background and the social norms of gender, women feel more obligated to provide care than men and feel more strain^45^.

Our study has several limitations. Although our analyses are based on a large group of patients, we cannot be confident that our cohort is representative. Most participants were members of the national self-help group representing approximately a quarter of all German MG patients. Moreover, we investigated only German MG patients. Although the Global Burden of Disease data from the World Health Organization (WHO) showed comparable prevalences amongst high-income countries like European Countries or the United States^46^, our data might not reflect the situation of MG patients in low-income countries. Importantly, due to the cross-sectional design of our study, we cannot conclude causality. Psychiatric disorders such as depression and anxiety disorders can be misdiagnosed as myasthenia gravis and vice versa. This is due to the overlapping complaints as well as the methodological limits of a patient survey. Thus, psychiatric comorbidities including PTSD may then also be captured too sensitively. Although the HADS is a reliable screening tool for assessing depression and anxiety, it is not identical to the ICD-10 diagnostic criteria and thus clinical diagnosis. The prevalence of depression based on HADS screening may therefore be overestimated^47^. Similarly, our results from the PTSD-7 questionnaire must be interpreted cautiously, as they cannot be equated with a PTSD diagnosis. Another limitation is our definition of myasthenic crisis. To distinguish between myasthenic crisis and exacerbation, we asked patients not only whether they had a "myasthenic crisis" but also whether they were treated in an intensive care unit setting in that case. For patients who reported a myasthenic crisis and were treated in an intensive care unit, we assumed that they had a myasthenic crisis. For patients who reported a myasthenic crisis and were treated in a normal ward setting, we assumed that they had an exacerbation. For assessing gender-related differences in QoL and disease burden we used a heteronormative approach, which does not consider non-binary gender as well as same-sex partnership. Finally, we used the MG-QoL15 scale and not yet the new slightly revised version (MG-QoL15r), which is preferred in current research because of its slightly better performance^48^.

## Conclusion

A higher awareness of mental illnesses like depression or anxiety is urgently required in MG care. Measures for depression, anxiety disorder, HRQoL, and activities of daily life should be standard tools in MG care. Female MG patients are at higher risk and might benefit from a systematic approach to mental health status assessment in particular. This strategy should be applied as early as possible in the course of the disease to offer appropriate treatment services, such as psychotherapeutic care in the outpatient setting. Our findings suggest that guideline-based treatment of depression can improve perceived illness severity and HRQoL in MG patients and reduce caregiver burden.

## Data Availability

The data that support this study’s findings are available on request from the corresponding author.

## References

[1] Verschuuren JJGM, Palace J, Gilhus NE (2010). Clinical aspects of myasthenia explained. Autoimmunity.

[2] Carr AS, Cardwell CR, McCarron PO, McConville J (2010). A systematic review of population based epidemiological studies in Myasthenia Gravis. BMC Neurol..

[3] Gold R, Hohlfeld R, Toyka KV (2008). Progress in the treatment of myasthenia gravis. Ther. Adv. Neurol. Disord..

[4] Thomas CE, Mayer SA, Gungor Y, Swarup R, Webster EA, Chang I (1997). Myasthenic crisis: clinical features, mortality, complications, and risk factors for prolonged intubation. Neurology.

[5] Boldingh MI, Dekker L, Maniaol AH, Brunborg C, Lipka AF, Niks EH (2015). An up-date on health-related quality of life in myasthenia gravis -results from population based cohorts. Health Qual. Life Outcomes.

[6] Basta IZ, Pekmezovic TD, Peric SZ, Kisic-Tepavcevic DB, Rakocevic-Stojanovic VM, Stevic ZD (2012). Assessment of health-related quality of life in patients with myasthenia gravis in Belgrade (Serbia). Neurol. Sci. Off. J. Ital. Neurol. Soc. Ital. Soc. Clin. Neurophysiol..

[7] Petersson M, Feresiadou A, Jons D, Ilinca A, Lundin F, Johansson R (2021). Patient-reported symptom severity in a nationwide myasthenia gravis cohort: Cross-sectional analysis of the Swedish GEMG study. Neurology.

[8] Qiu L, Feng H, Huang X, Mo R, Ou C, Luo C (2010). Study of incidence and correlation factors of depression, anxiety and insomnia in patients with myasthenia gravis. Zhonghua Yi Xue Za Zhi.

[9] Suzuki Y, Utsugisawa K, Suzuki S, Nagane Y, Masuda M, Kabasawa C (2011). Factors associated with depressive state in patients with myasthenia gravis: A multicentre cross-sectional study. BMJ Open.

[10] Twork S, Wiesmeth S, Klewer J, Pöhlau D, Kugler J (2010). Quality of life and life circumstances in German myasthenia gravis patients. Health Qual. Life Outcomes.

[11] Winter Y, Schepelmann K, Spottke AE, Claus D, Grothe C, Schröder R (2010). Health-related quality of life in ALS, myasthenia gravis and facioscapulohumeral muscular dystrophy. J. Neurol..

[12] Law C, Flaherty CV, Bandyopadhyay S (2020). A review of psychiatric comorbidity in Myasthenia gravis. Cureus.

[13] Kanner AM (2005). Should neurologists be trained to recognize and treat comorbid depression of neurologic disorders? Yes. Epilepsy Behav..

[14] Hinz A, Schwarz R, Herrmann C, Buss U, Snaith R (2002). Hospital anxiety and depression scale - Deutsche Version(HADS-D). Diagnostica.

[15] Burns TM, Grouse CK, Wolfe GI, Conaway MR, Sanders DB (2011). The MG-QOL15 for following the health-related quality of life of patients with myasthenia gravis. Muscle Nerve.

[16] Breslau N, Peterson EL, Kessler RC, Schultz LR (1999). Short screening scale for DSM-IV posttraumatic stress disorder. Am. J. Psychiatry.

[17] Maercker A, Forstmeier S, Wagner B, Glaesmer H, Brähler E (2008). Posttraumatische Belastungsstörungen in Deutschland. Nervenarzt.

[18] Muthny FA, Verres R (1989). Freiburger Fragebogen zur Krankheitsverarbeitung. Psychosoz Onkol.

[19] Graessel E, Berth H, Lichte T, Grau H (2014). Subjective caregiver burden: validity of the 10-item short version of the Burden scale for family caregivers BSFC-s. BMC Geriatr..

[20] Doering S, Henze T, Schussler G (1993). Coping With Myasthenia Gravis and implications for psychotherapy. Arch Neurol..

[21] Hoffmann S, Ramm J, Grittner U, Kohler S, Siedler J, Meisel A (2016). Fatigue in myasthenia gravis: Risk factors and impact on quality of life. Brain Behav..

[22] Ybarra M, Kummer A, Comini-Frota E, Oliveira J, Gomez R, Teixeira A (2011). Psychiatric disorders in myasthenia gravis. Arq. Neuropsiquiatr..

[23] Lundeen J, Fisher J, Kothari MJ (2004). Frequency of anxiety in myasthenia gravis. J. Clin. Neuromuscul. Dis..

[24] Liu C, Li T, Wang Q, Xu A, Wu B (2020). Post-traumatic stress disorder symptoms after respiratory insufficiency in patients with myasthenia gravis. Psychol. Health Med..

[25] Jacobi F, Höfler M, Strehle J, Mack S, Gerschler A, Scholl L (2014). Psychische Störungen in der Allgemeinbevölkerung. Studie zur gesundheit erwachsener in Deutschland und ihr zusatzmodul psychische gesundheit (DEGS1-MH). Nervenarzt..

[26] Rafsten L, Danielsson A, Sunnerhagen KS (2018). Anxiety after stroke: A systematic review and meta-analysis. J. Rehabil. Med..

[27] Pérez-Piñar M, Ayerbe L, González E, Mathur R, Foguet-Boreu Q, Ayis S (2017). Anxiety disorders and risk of stroke: A systematic review and meta-analysis. Eur. Psychiatry.

[28] Boeschoten RE, Braamse AMJ, Beekman ATF, Cuijpers P, van Oppen P, Dekker J (2017). Prevalence of depression and anxiety in multiple sclerosis: A systematic review and meta-analysis. J. Neurol. Sci..

[29] Rogers SC (2017). Poststroke depression screening: An executive summary. J. Neurosci. Nurs. J. Am. Assoc. Neurosci. Nurses.

[30] Kulaksizoglu IB (2007). Mood and anxiety disorders in patients with myasthenia gravis: Aetiology, diagnosis and treatment. CNS Drugs.

[31] Perez-Nellar J, Rodriguez A (2000). False negatives in the diagnosis of myasthenia gravis. Rev. Neurol..

[32] Rohr W (1992). Myasthenia gravis in the diagnostic frontier area of psychiatry. Psychiat. Prax..

[33] Braz NFT, Rocha NP, Vieira ÉLM, Barbosa IG, Gomez RS, Kakehasi AM (2018). Muscle strength and psychiatric symptoms influence health-related quality of life in patients with myasthenia gravis. J. Clin. Neurosci. Off. J. Neurosurg. Soc. Australas.

[34] Lee I, Kaminski HJ, Xin H, Cutter G (2018). Gender and quality of life in myasthenia gravis patients from the myasthenia gravis foundation of America registry. Muscle Nerve.

[35] Bogdan A, Barnett C, Ali A, AlQwaifly M, Abraham A, Mannan S (2020). Chronic stress, depression and personality type in patients with myasthenia gravis. Eur. J. Neurol..

[36] Fan X, Xing C, Yang L, Wang J, Feng L (2020). Fatigue, self-efficacy and psychiatric symptoms influence the quality of life in patients with myasthenia gravis in Tianjin, China. J. Clin. Neurosci. Off. J. Neurosurg. Soc. Australas.

[37] Kroenke K, Spitzer RL (1998). Gender differences in the reporting of physical and somatoform symptoms. Psychosom. Med..

[38] Sacks CR, Peterson RA, Kimmel PL (1990). Perception of illness and depression in chronic renal disease. Am. J. Kidney Dis..

[39] Murphy H, Dickens C, Creed F, Bernstein R (1999). Depression, illness perception and coping in rheumatoid arthritis. J. Psychosom. Res..

[40] Steca P, Greco A, Monzani D, Politi A, Gestra R, Ferrari G (2013). How does illness severity influence depression, health satisfaction and life satisfaction in patients with cardiovascular disease? The mediating role of illness perception and self-efficacy beliefs. Psychol. Health.

[41] Wolfe GI, Kaminski HJ, Aban IB, Minisman G, Kuo H-C, Marx A (2016). Randomized trial of thymectomy in myasthenia gravis. N. Engl. J. Med..

[42] Rückert JC, Swierzy M, Kohler S, Meisel A, Ismail M (2018). Thymektomie bei myasthenia gravis TT - thymectomy in Myasthenia gravis. Aktuelle Neurol..

[43] Sherwood PR, Given CW, Given BA, von Eye A (2005). Caregiver burden and depressive symptoms: Analysis of common outcomes in caregivers of elderly patients. J. Aging Health.

[44] Miech RA, Shanahan MJ (2000). Socioeconomic status and depression over the life course. J. Health Soc. Behav..

[45] Bjelland I, Krokstad S, Mykletun A, Dahl A, Tell G, Tambs K (2008). Does higher education protect against anxiety and depression? The HUNT study. Soc. Sci. Med. (1982).

[46] James SL, Abate D, Abate KH, Abay SM, Abbafati C, Abbasi N (2018). Global, regional, and national incidence, prevalence, and years lived with disability for 354 diseases and injuries for 195 countries and territories, 1990–2017: A systematic analysis for the Global Burden of disease study 2017. Lancet.

[47] Stoppe, G., Bramesfeld, A., Schwartz, F.-W. (2006) Volkskrankheit Depression? Bestandsaufnahme und Perspektiven. *Dtsch. Arztebl. Int.* **103(22),** A–1557. Available from: https://www.aerzteblatt.de/int/article.asp?id=51635.

[48] Burns TM, Sadjadi R, Utsugisawa K, Gwathmey KG, Joshi A, Jones S (2016). International clinimetric evaluation of the MG-QOL15, resulting in slight revision and subsequent validation of the MG-QOL15r. Muscle Nerve.

